# An Italian real-world multicenter study of patients with refractory/relapsed functional high-risk multiple myeloma patients treated with second-line therapies

**DOI:** 10.1007/s00277-025-06572-y

**Published:** 2025-09-10

**Authors:** Danilo De Novellis, Salvatore Palmieri, Stefano Rocco, Daniele Derudas, Roberta Della Pepa, Daniela Roccotelli, Daniela Esposito, Chiara Masucci, Emilia Gigliotta, Maria Lucia Barone, Emanuela Morelli, Antonio Lazzaro, Rosario Bianco, Fabrizio Accardi, Luana Marano, Matteo Bonanni, Anna Maria Della Corte, Bianca Serio, Eleonora Urciuoli, Michela Rizzo, Raffaele Fontana, Manuela Arcamone, Rossella Iula, Anna Dandolo, Aldo Leone, Maria Gabriella Rascato, Maria Di Perna, Antonietta Pia Falcone, Lucia Morello, Gianpaolo Marcacci, Nunziata Giuseppe Rodolfo, Ferdinando Frigeri, Catello Califano, Angelo Michele Carella, Antonio Maria Risitano, Mario Annunziata, Fabrizio Pane, Valentina Giudice, Cirino Botta, Carmine Selleri

**Affiliations:** 1 Hematology and Transplant Center, University Hospital“San Giovanni di Dio e Ruggi d’Aragona”, Salerno, Italy; 2 Department of Medicine, Surgery and Dentistry“Scuola Medica Salernitana”, University of Salerno, Baronissi, Italy; 3 Hematology, Hospital “Antonio Cardarelli”, Naples, Italy; 4 S.C. di Ematologia e C.T.M.O, Ospedale Oncologico di Riferimento Regionale“A. Businco”, Cagliari, Italy; 5 Hematology - Department of Clinical Medicine and Surgery, University Hospital “Federico II”, Naples, Italy; 6 Division of Hematology and Stem Cell Unit, IRCCS S. Giovanni Rotondo and Division of Hematology, San Giovanni Rotondo, Italy; 7 Hematology, Hospital “San Giuseppe Moscati”, Aversa, Italy; 8 Hematology, Hospital “San Giuseppe Moscati”, Avellino, Italy; 9 Dipartimento di Promozione della Salute, Materno-infantile, di medicina interna e specialistica d’eccellenza “G. D’alessandro”, Università di Palermo, Palermo, Italy; 10 Hematology, Hospital “Andrea Tortora”, Pagani, Italy; 11 Hematology and Transplant Center, Istituto Nazionale Tumori,fondazione “G.Pascale”, IRRCS, Naples, 80131 Italy; 12 Ematologia e Centro Trapianti Midollo Osseo, Ospedale Guglielmo da Saliceto, Piacenza, Italy; 13 Hematology, Hospital “Sant’Anna e San Sebastiano”, Caserta, Italy; 14 U.O.C di Oncoematologia, Azienda Ospedaliera Ospedali Riuniti Villa Sofia - Cervello, Palermo, Italy; 15 Department of Medicine, Surgery and Dentistry “Scuola Medica Salernitana” University Hospital “San Giovanni di Dio e Ruggi d’Aragona”, University of Salerno, Baronissi, 84081 Italy

**Keywords:** Multiple myeloma, High functional risk, Quadruplets, Real-life

## Abstract

Functional high risk multiple myeloma (FHRMM) remains a challenging entity with poor outcomes and limited survival, and there is no international consensus on optimal second-line therapeutic strategies in relapsed/refractory patients. In this multicenter real-world retrospective study, we investigated clinical characteristics and outcomes of a total of 62 FHRMM patients previously treated with a first-line daratumumab-based quadruplet regimen or who relapsed within 12 months after frontline autologous stem cell transplantation (ASCT). In our cohort, the overall response rate was 61%, with 42% of patients achieving a very good partial response (VGPR) or better. Similarly, median progression-free survival (PFS) was not reached with an estimated 12-month PFS rate of 54%, as well as median overall survival (OS) with a 12-month OS rate of 72%. Factors associated with worse PFS included extramedullary disease, prior lenalidomide maintenance, lack of ASCT consolidation, an ECOG score ≥ 2, advanced disease stage, and salvage therapy without carfilzomib-lenalidomide-dexamethasone. In conclusion, second-line management of FHRMM following daratumumab-bortezomib-thalidomide-dexamethasone induction is highly challenging and variable across centers, due to the lack of standardized international guidelines. Carfilzomib-based regimens demonstrated some clinical benefits, especially in lenalidomide-naïve patients; however, outcomes remained suboptimal in FHRMM population who may benefit from novel therapies administered as earlier treatment lines. Larger prospective trials are needed to optimize FHRMM clinical management and improve patient outcomes.

**Supplementary Information:**

The online version contains supplementary material available at 10.1007/s00277-025-06572-y.

## Introduction

Multiple myeloma (MM) is a hematological malignancy arising from uncontrolled proliferation of neoplastic bone marrow plasma cells (PCs), leading to the overproduction of monoclonal complete immunoglobulins or light chain and their accumulation in tissues and organs, resulting in end-organ damage [1, 2]. Clinical history of MM is marked by alternating periods of remission following induction therapy and subsequent relapses, with patients requiring multiple lines of treatment and often developing refractoriness to previously used drug classes [1, 3]. After the introduction of anti-CD38 monoclonal antibodies with first line regimens, prognosis and survival have significantly improved. CD38, a single chain type II transmembrane glycoprotein, is highly expressed on both normal and neoplastic PCs, while exhibiting low expression levels in non-PC populations such as myeloid cells, making it an ideal therapeutic target in MM [3]. In the multicenter randomized phase III CASSIOPEIA trial [4], patients were randomly assigned to receive induction therapy with bortezomib-thalidomide-dexamethasone (VTD) with or without daratumumab, the first-in-class anti-CD38 antibody. Daratumumab-treated patients showed remarkable outcomes, with stringent complete response (sCR) rates of 29% and an overall response rate (ORR) of 92.6%, resulting in a significant improvement in median progression free survival (PFS) [4, 5]. However, some patients remain primary refractory or experience early relapse, even after daratumumab-VTD (Dara-VTD) induction or autologous stem cell transplantation (ASCT).

Functional high-risk multiple myeloma (FHRMM) is a dynamic classification based on disease kinetics, wherein patients are stratified according to their intrinsic resistance to induction treatments. This category includes primary refractory and early relapsed diseases occurring within 18 months from treatment initiation or within 12 months from frontline ASCT, despite optimal first-line therapy [6]. Unlike conventional high-risk MM classifications based on genetic abnormalities or other adverse prognostic markers, FHRMM is defined primarily by clinical behavior, although genetic characteristics may contribute to its aggressive phenotype [7]. FHRMM patients face an extremely poor prognosis, yet limited data exist on the outcomes of daratumumab-treated populations and following early relapse or refractoriness after Dara-VTD induction [8–11].

In this retrospective observational multicenter real-life Italian study, we aimed to describe second-line therapeutic strategies for FHRMM after Dara-VTD induction, and to assess the overall efficacy and safety of these treatment approaches. Moreover, we investigated the impact of different factors on survival outcomes, seeking to identify key risk factors associated with disease relapse and progression.

## Patients and methods

### Study cohort

A total of 62 consecutive FHRMM patients from 13 Italian Hematology Units who initiated second-line therapy outside clinical trials after Dara-VTD induction were enrolled from January 2022 to November 2024. Inclusion criteria were: age *≥* 18 years; diagnosis of MM based on 2016 International Myeloma Working Group (IMWG) updated criteria [12]; stable or progressive disease, or relapse within 18 months from treatment initiation or within 12 months from frontline ASCT following Dara-VTD induction, according to IMWG response criteria [12]; and administration of any second-line anti-MM therapy. High-risk genetic abnormalities were assessed according to IMWG criteria, with the addition of chromosome 1q gain/amplification and 1p deletion [12]. Daratumumab refractoriness was defined as progressive disease or no response during therapy or within 60 days of stopping treatment according to the International Uniform Response Criteria for Multiple Myeloma. The decision to proceed with salvage ASCT was based on clinical assessment, center guidelines, and the type of hematologic response achieved on second-line therapy.

This study was conducted in accordance with the Declaration of Helsinki, the International Conference on Harmonization Good Clinical Practice guidelines [13], and protocols approved by our Ethics Committee “Campania Sud”, Brusciano, Naples, Italy (prot./SCCE n. 24988). All patients provided written informed consent.

### Endpoints

Primary endpoint was PFS, defined as the time from second line therapy initiation to the first documented disease progression or death. Secondary endpoints included: ORR, stringent complete response (sCR), CR, very good partial response (VGPR), and partial response (PR), as defined according to IMWG criteria [14]; overall survival (OS); time to best response; duration of response (DoR); and safety, assessed using the National Cancer Institute’s Common Terminology Criteria for Adverse Events version 6.0 (CTCAE v6.0). Laboratory assessments were evaluated at baseline, before each treatment cycle, and at treatment discontinuation. Antibiotic, antiviral, and antifungal prophylaxis were administered according to international guidelines [15, 16].

### Statistical analysis

Data were collected in spreadsheets and analyzed using R statistical software (v. 4.0.5; RStudio), and SPSS (v. 25; IBM). Comparisons between groups were performed by Chi-square, Fisher’s, Wilcoxon signed-rank, or unpaired two-tailed t-tests, where appropriate. Kaplan-Meyer, log-rank, and Breslow tests were used for survival analysis. Univariate Cox regression models were applied to examine effects (hazard ratio; HR) of independent variables on survival. Response monitoring for each patient was displayed using a Sankey diagram. A *P*-value of < 0.05 was considered statistically significant.

## Results

### Clinical characteristics and therapeutic history at second line treatment initiation

A total of 62 patients (median age, 60 years old; range, 40–71 years old; M/F, 34/28) were included in this study, with a median follow-up of 14 months at data cut-off, and an Eastern Cooperative Oncology Group (ECOG) performance status of 0–1 in 49 subjects (79%) and ≥ 2 in 11 (14%). Clinical, laboratory, and biological features at second-line initiation are summarized in Table 1. Monoclonal proteins were IgG in 42 patients (68%), IgA in nine (14%), and light chain in 11 (19%) cases. Light chain type was κ or λ in 42 (68%) and 20 patients (32%), respectively. High genetic risk was present in 28 patients (45%), with 6 cases (10%) carrying ≥ 2 high risk abnormalities, severe renal failure, defined as glomerular filtration rate (GFR) ≤ 40 ml/min, in 11 subjects (18%), and extramedullary disease (EMD) in 17 cases (27%). In particular, EMD was bone-associated in 5 (8%) and bone-independent in 12 cases (19%). Median LDH was 207 mUI/mL (range, 73-4743 mUI/mL), median β2-microglobulin levels were 3.3 mg/dL (range, 1.3–26 mg/dL), and median albumin levels were 2.8 g/dL (range, 2–4.1 g/dL). According to the revised International Staging System (R-ISS), three patients (4%) were classified as stage I, 39 (63%) as stage II, and 20 (33%) as stage III. Comorbidities were present in 15 patients (24%) and included hypertension (*N* = 5; 8%), cardiac ischemic disease (*N* = 3; 5%), prior solid neoplasia (*N* = 2; 3%), obesity (*N* = 2; 3%), type II diabetes (*N* = 2; 3%), and Tako-Tsubo syndrome (*N* = 1; 1%).

**Table 1:** Patients’ characteristics at enrollment

Characteristics	*N* = 62
Median age, years (range)	60 (40–71)
Male, n (%)	34 (55)
ECOG scale, n (%) – 0–1 – ≥ 2	49 (79) 11 (14)
M-protein type, n (%) – IgG – IgA – Light chain only	42 (68) 9 (14) 11 (18)
Light chain κ/λ, n (%)	42 (68)/20 (32)
Glomerular filtration rate ≤ 40 ml/min, n (%)	11 (18)
High genetic risk MM*, n (%)	28 (45)
Number of high genetic risk abnormalities, n (%) 1 ≥ 2	22 (35) 6 (10)
EMD, n (%) – Bone-associated – Soft tissue	17 (27) 5 (8) 12 (19)
Median LDH, mU/mL (range)	207 (73-4743)
Median albumin, gr/dL (range)	2.8 (2-4.1)
Median β2-microglobulin, mg/dL (range)	3.3 (1.3–26)
Revised international staging system, n (%) – I-II – III	42 (67) 20 (33)
Dara-VTD cycles before second line, n (%) – 1–2 – 3–4 – 5–6	5 (8) 41 (67) 16 (25)
Median time of dara-VTD exposure, months (range)	4 (1–8)
Prior ASCT, single/double, n (%)	11 (18)/2 (3)
Prior lenalidomide maintenance, n (%) Lenalidomide refractory, n (%)	4 (6) 4 (6)
Median time from ASCT, months (range)	4.5 (1–11)
Primary refractory disease, n (%) Early relapsed disease, n (%) Daratumumab refractory, n (%)	47 (76) 15 (24) 54 (87)
Comorbidities, n (%) – Hypertension – Cardiac ischemia – Previous solid neoplasia – Obesity – Type II diabetes – Tako-Tsubo syndrome	15 (24) 5 (8) 3 (5) 2 (3) 2 (3) 2 (3) 1 (1)

Abbreviations. *ECOG* Eastern Cooperative Oncology Group, *Ig* immunoglobulin, *MM* multiple myeloma, *EMD* extramedullary disease, *LDH* lactate dehydrogenase, *Dara-VTD* daratumumab-bortezomib-thalidomide-dexamethasone, *ASCT* autologous stem cell transplantation

*High genetic risk MM was defined according to IMWG criteria, with the addition of chromosome 1q gain/amplification and 1p deletion

In our cohort at the time of data cut-off, 5 patients (8%) received 1–2 Dara-VTD cycles, 41 (67%) 3–4 cycles, and 16 subjects (25%) 5–6 cycles, with a median therapy exposure of 4 months (range, 1–8 months). Primary refractoriness and early relapses occurred in 47 (76%) and 15 (24%) patients, respectively, and 54 (87%) of them were considered daratumumab refractory according to IMWG definition. Among early relapsed cases, 13 subjects (21%) previously underwent ASCT, with 11 of them (18%) receiving a single ASCT and two (3%) tandem transplantation, and four of them (6%) also initiated lenalidomide maintenance, who were considered refractory to lenalidomide at the time of MM progression. Median time from prior ASCT to second-line therapy initiation was 4.5 months (range, 1–11 months).

### Second-line therapies and treatment outcomes

**Fig. 1:**
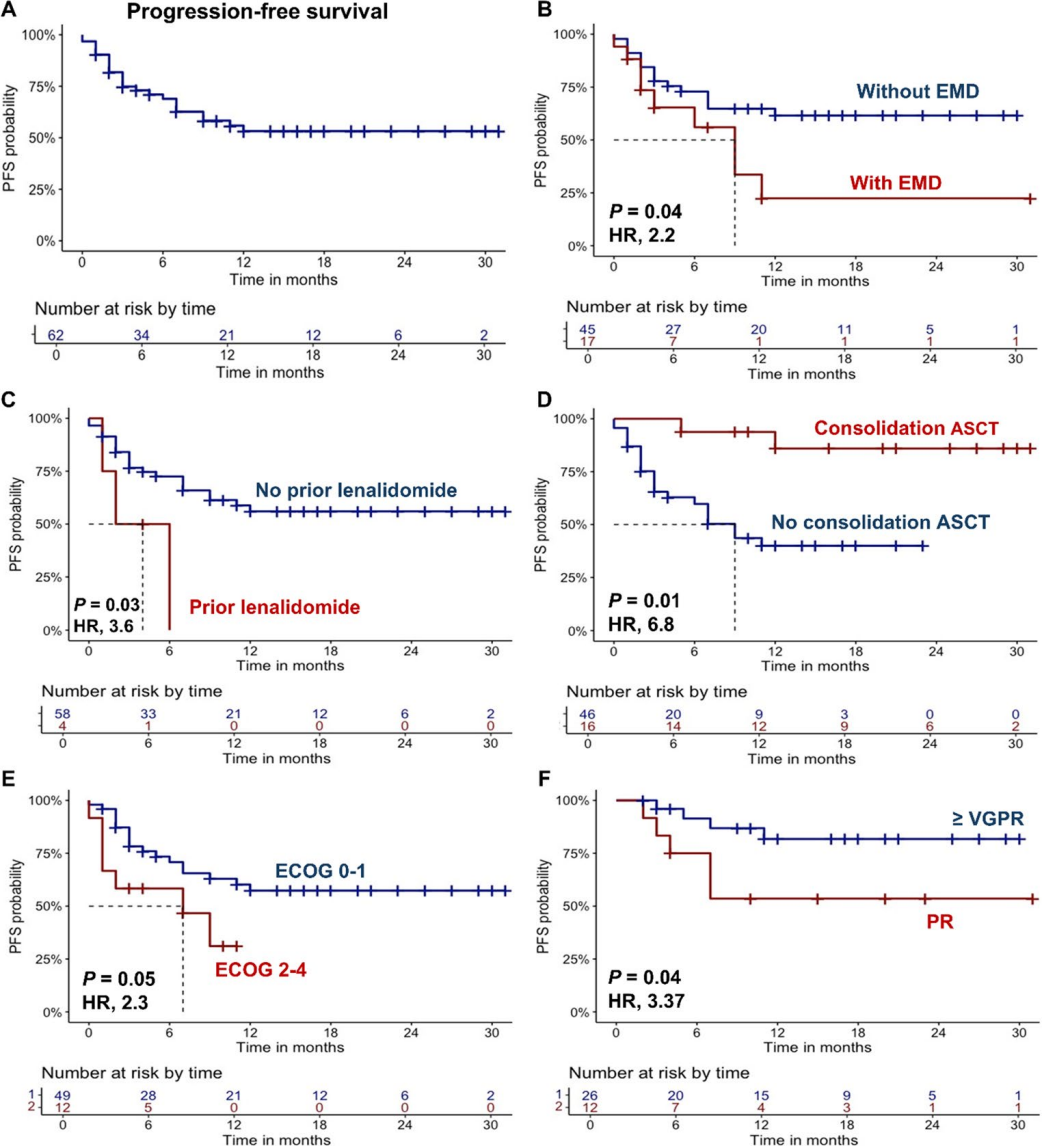
Progression-free survival (PFS) in functional high risk multiple myeloma patients treated with second-line therapies. **A** PFS is shown for the entire cohort of patients, and after stratification for **B** extramedullary disease (EMD), **C** prior lenalidomide administration or **D** autologous stem cell transplantation (ASCT) consolidation, **E** ECOG status, or **F** very good partial response (VGPR) or partial response (PR) achievement. A *P* < 0.05 was considered statistically significant. HR, hazard ratio

**Fig. 2:**
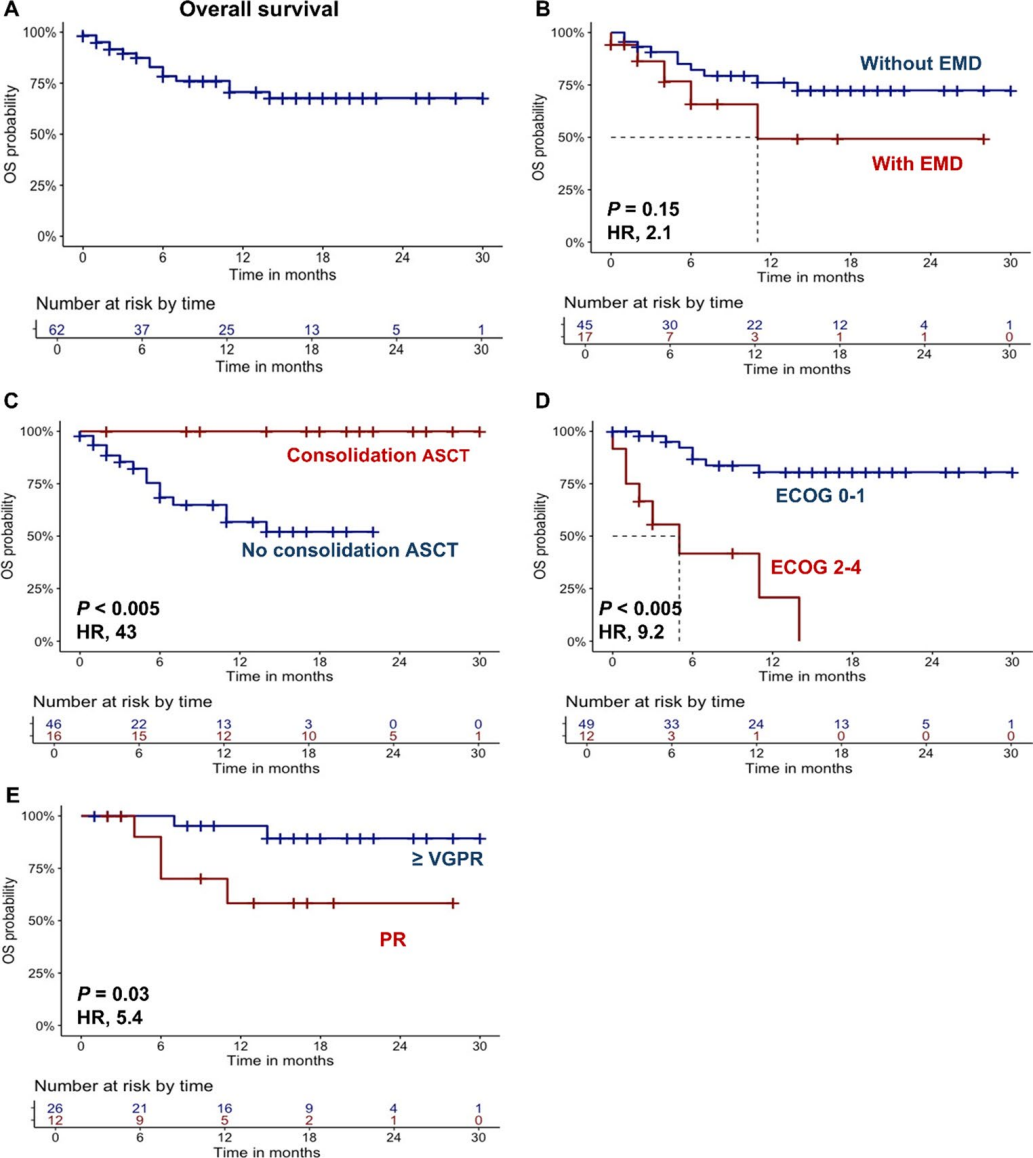
Overall survival (OS) in functional high risk multiple myeloma patients treated with second-line therapies. **A** OS is shown for the entire cohort of patients, and after stratification for **B** extramedullary disease (EMD), **C** prior autologous stem cell transplantation (ASCT) consolidation, **D** ECOG status, or **E** very good partial response (VGPR) or partial response (PR) achievement. A *P* < 0.05 was considered statistically significant. HR, hazard ratio

In our cohort, carfilzomib-lenalidomide-dexamethasone (KRD) was the most used second-line treatment, administered to 53 patients (86%), while chemotherapy, including D-PACE or PAD regimens, were employed in 5 cases (8%) (Table 2). Median PFS was not reached (NR; Fig. 1A) with an estimated 12-month PFS of 54%, as well as median OS was NR, with a 12-month OS of 72% (Fig. 2A). The ORR was 61% (*N* = 38), with 8 subjects (13%) in CR or sCR, 18 (29%) in VGPR, and 12 (19%) in PR, while a VGPR or better response was observed in 26 (42%) patients. The median time to best response was 2 months (range, 1–24 months) and the median number of treatment cycles was 4 (range, 1–30). Consolidation with ASCT was performed in 16 (26%) cases who did not receive transplantation before second-line therapy. At data cut-off, treatment was still ongoing in 21 cases (34%), while it was discontinued in the remaining subjects for the following reasons: disease progression (*N* = 24; 39%); ASCT or stem cell collection (*N* = 6; 10%); death (*N* = 4; 6%); clinical decision (*N* = 4; 6%); unacceptable toxicity (*N* = 2, 3%); or patient’s choice (*N* = 1; 1%). Fifteen (24%) patients died, mainly due to disease progression (*N* = 11; 73%) or to infectious complications (*N* = 4; 27%).

**Table 2:** Clinical outcomes after second-line therapies

Outcomes	*N* = 62
Type of second line therapy, n (%) – KRD – Chemotherapy – Other	53 (86) 5 (8) 4 (6)
ORR, n (%) – CR, n (%) – VGPR, n (%) – PR, n (%) – VGPR or better, n (%)	38 (61) 8 (13) 18 (29) 12 (19) 26 (42)
Median time-to-best response, months (range)	2 (1–24)
Consolidation with ASCT, n (%)	16 (26)
Overall progressions after second line therapy, n (%)	24 (39)
Median PFS, months (range)	NR (1–27)
12-month PFS, %	54
Median OS, months (range)	NR (2–28)
12-month OS, %	72
Deaths, n (%) – Progression – Infections	15 (24) 11 (73) 4 (27)

Abbreviations. *KRD* carfilzomib-lenalidomide-dexamethasone, *ORR* overall response rate, *CR* complete response, *VGPR* very good partial response, *PR* partial response, *ASCT* autologous stem cell transplantation, *PFS* progression-free survival, *OS* overall survival

Next, patients were divided based on clinical characteristics, and clinical outcomes were compared. In details, patients with EMD showed reduced median PFS compared to those without EMD (9 months vs. NR; HR, 2.2; 95% confidential interval [CI], 1.2-5; *P* = 0.04), as well as patients who had received lenalidomide maintenance (4 months vs. NR; HR, 3.6; 95%CI, 1.1–12.4; *P* = 0.03), or those who did not undergo ASCT consolidation (9 months vs. NR; HR, 6.8; 95%CI, 1.6–28.9; *P* = 0.01). In addition, patients with ECOG ≥ 2 also displayed a shorter PFS compared to fitter subjects (7 months vs. NR; HR, 2.3; 95%CI, 1.1–5.6; *P* = 0.05), as well as patients who did not achieve at least a VGPR (12 month-PFS, 53% vs. 82%; HR, 3.37; 95%CI, 1.2–12; *P* = 0.04) (Fig. 1B-F). No significant differences in PFS were observed when patients were divided based on prior ASCT (*P* = 0.81), time since prior ASCT (*P* = 0.8), age > 60 years (*P* = 0.6), high genetic risk (*P* = 0.8), female sex (*P* = 0.06), KRD regimen administration (*P* = 0.5), or higher R-ISS stages (*P* = 0.4) (Supplementary Fig. 1).

**Fig. 3:**
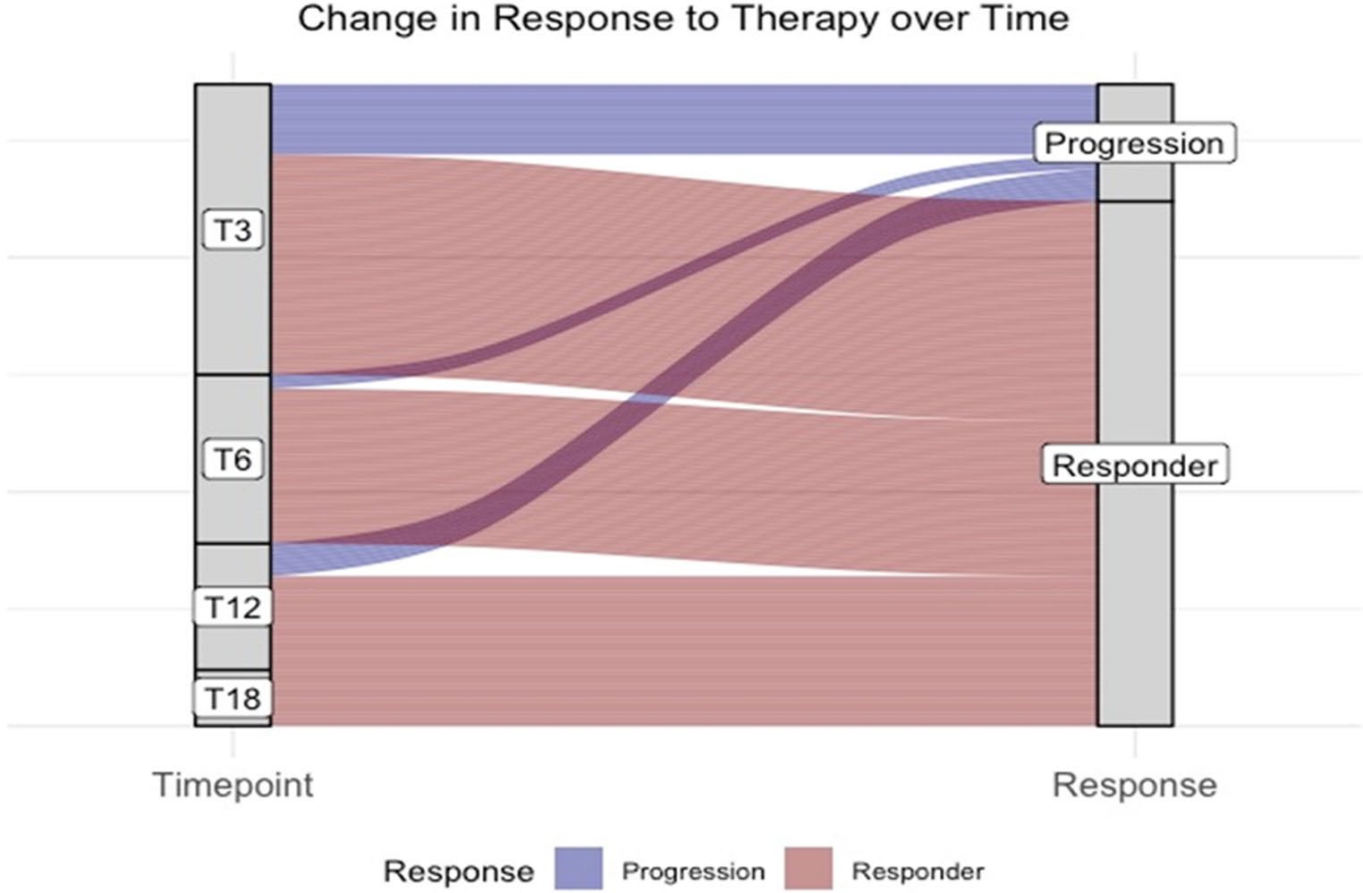
Progression rates. Change in response to therapy at different time points are shown at three (T3), six (T6), 12 (T12), and 18 months (T18) from diagnosis in functional high risk multiple myeloma patients

Similarly, patients with EMD also showed a reduced OS (median OS, 11 months vs. NR; HR, 2.1; 95%CI, 0.85–6.1; *P* = 0.15), as well as those without consolidation with ASCT (12-month OS, 56% vs. 100%; HR, 43; 95CI, 1.5–359; *P* < 0.005), or an ECOG ≥ 2 (5 months vs. NR; HR, 9.2; 95%CI, 3.2–26.1; *P* < 0.005), or those who did not achieve at least a VGPR (12-month OS, 58% vs. 95%; HR, 5.4; 95%CI, 1.3–29.7; *P* = 0.03) (Fig. 2B-F). No significant differences were observed when patients were divided based on female sex (*P* = 0.4), high genetic risk (*P* = 0.54), KRD regimen administration (*P* = 0.8), R-ISS stage (*P* = 0.8), prior ASCT (*P* = 0.9), time since prior ASCT (*P* = 0.9), or age > 60 years (*P* = 0.5) (Supplementary Fig. 2). Furthermore, no differences in PFS or OS were observed according to the presence or absence of refractoriness to daratumumab (data not shown). Finally, late progressions after 12 months were not observed (*N* = 15 at three months; *N* = 3 at six months; *N* = 7 at twelve months; and *N* = 0 at 18 months) (Fig. 3).

### Toxicity and infections

Hematological toxicity was documented in 25 patients (40%), with grade I-II anemia, thrombocytopenia or neutropenia in 14 (23%) subjects, and grade III-IV hematological toxicities in 11 cases (17%). Cardiac adverse events occurred in 10 patients (16%), including grade I-II and III-IV hypertension (*N* = 6, 11%; and *N* = 1, 1%; respectively), tachyarrhythmias (*N* = 2; 3%), and Tako-Tsubo syndrome (*N* = 1; 1%). Infections were observed in 9 patients (15%), and included upper airway infections (*N* = 4; 6%), pneumonia (*N* = 3; 5%), or bacteriemia (*N* = 2; 3%).

## Discussion

In recent years, the inclusion of novel anti-MM agents, such as anti-CD38 monoclonal antibodies, in first-line therapies has significantly improved clinical outcomes of MM patients. However, a small fraction of patients still fail to achieve a hematological response due to simultaneous and unexpected refractoriness to proteasome inhibitors, immunomodulatory drugs, and anti-CD38 monoclonal antibodies. The clinical outcomes of triple-class refractory MM patients remain extremely poor, with an ORR of 29.8% and a median PFS of 4.6 months in multi-treated subjects (median of four prior therapy lines; range, 1–20), as shown in the LocoMMotion trial [17]. In particular, these subjects are now categorized as FHRMM, which is a dynamic concept of the disease, where patients are either primary refractory or experience early relapse after an optimal first-line induction, for unknown reasons that are not necessarily linked to high-risk genetic abnormalities. Therefore, FHRMM patients have poor prognosis, also in real-world settings [8–10], although these studies have been conducted before the introduction of daratumumab-based quadruplet regimens in routine clinical practice. In our study, we reported the first real-world evidence of efficacy of second-line therapies in FHRMM patients previously treated with Dara-VTD induction, including in our cohort only those patients who have received only one prior line of therapy and were already triple-class refractory, in contrast to findings reported in the previous LocoMMotion trial.

Risk factors for FHRMM are still unknown, although EMD, characterized by aberrant plasma cell proliferation outside the bone marrow niche, is a well-established negative prognostic factor in MM patients [18, 19], slightly mitigated by daratumumab administration [20, 21]. In our real-life FHRMM cohort, EMD was associated with poorer outcomes, while other high-risk disease factors were not significantly related to shorter PFS or OS. Moreover, patients achieving VGPR or better showed a significant survival benefit with a plateau in the PFS curve in sustained responders, suggesting the importance to reaching an early and deep response after Dara-VTD failure, to improve clinical outcomes of FHRMM patients. In this view, consolidation with ASCT after second-line re-induction should be considered as an effective approach, despite the potential bias that only patients who achieved sustained and deep remissions are eligible to ASCT.

These data were also confirmed by our separate analysis of MM progression, where no events were observed after 12 months after the therapy started.

Carfilzomib, a potent second-generation proteasome inhibitor, irreversibly binds to the active sites of the 20 S proteasome and the 26 S proteasome core component, effectively targeting MM cells [22]. Inclusion of carfilzomib within the KRD regimen has resulted in improved median PFS compared to lenalidomide and dexamethasone alone (26.3 vs. 17.6 months) in relapsed/refractory MM patients, as documented in the randomized phase III ASPIRE trial [23]. In our study, KRD combination was the most preferred and effective treatment option, although our results were lower than those reported in the ASPIRE trial, likely due to the exclusive inclusion in our cohort of only FHRMM. Anti-CD38-based therapies were not chosen as second-line therapy, even for those patients who were not strictly refractory to daratumumab according to IMWG guidelines, as physicians preferred a change in drug class. Moreover, our patients who had maintenance therapy with lenalidomide and did not access a lenalidomide-based regimen as second-line therapy showed worse outcomes. Therefore, KRD could represent a valid second-line therapeutic option for these high-risk patients following anti-CD38-based induction and/or lenalidomide exposure.

However, early relapses or refractoriness to anti-CD38-based induction regimens, including Dara-VRD or Dara-KRD, remain still associated with poorer outcomes, with a median PFS of 2.5-7.0 months in patients who experience disease progression, regardless of T-cell redirecting therapy administration [24]. Conversely, more recent results from the KarMMA-2 study cohort 2B and the CARTITUDE-4 study subgroup analysis have shown that CAR-T products ide-cel and cilta-cel can improve the prognosis of FHRMM patients compared to the standard of care [25, 26]. Therefore, CAR-T therapies are a promising approach for FHRMM treatment; however, further evidence is needed to define the standard of care of these patients. For example, utilization of daratumumab or isatuximab plus VRD as first-line therapies for both ASCT-eligible and ineligible patients could maximize efficacy, reducing the risk of developing FHRMM and thus improving overall patient outcomes [27–29]; however, the presence of lenalidomide in these combinations could not allow to use this drug as salvage therapy in refractory patients.

Our study has several limitations: (i) its retrospective, real-world nature; (ii) a small sample size, limiting specific subgroup analyses, particularly for non-KRD regimens; (iii) the lack of bone marrow minimal residual disease (MRD) data, as systematic MRD monitoring is not yet recommended in real-world settings outside clinical trials; and (iv) potential variability in treatment approaches due to the study’s multicenter design.

In conclusion, we report the first multicenter real-life study investigating the efficacy and safety of second-line therapies in FHRMM following Dara-VTD induction, as well as clinical outcomes in a real-world setting. We showed that these patients experienced poor outcomes, although KRD could represent a valid option, especially in lenalidomide-naïve patients, although outcomes remain suboptimal. Therefore, there is an urgent unmeet clinical need to integrate bispecific agents and CAR-T therapies in earlier treatment lines, and to include in risk stratification systems additional molecular and phenotypic biomarkers to improve prognostic definition of these high-risk disease patients.

## Supplementary Information

Below is the link to the electronic supplementary material.

ESM 1 (PDF 1.20 MB)

## Data Availability

No datasets were generated or analysed during the current study.
