# Supplementary material for: An Italian real-world multicenter study of patients with refractory/relapsed functional high-risk multiple myeloma patients treated with second-line therapies
Source: Ann Hematol. 2025 Sep 10;104(9):4749–57. doi: 10.1007/s00277-025-06572-y (PMC12552284; doi:10.1007/s00277-025-06572-y)
Supplement: Supplementary file 1 — (PDF 1.20 MB) [file 277_2025_6572_MOESM1_ESM.pdf]

Supplementary Informations

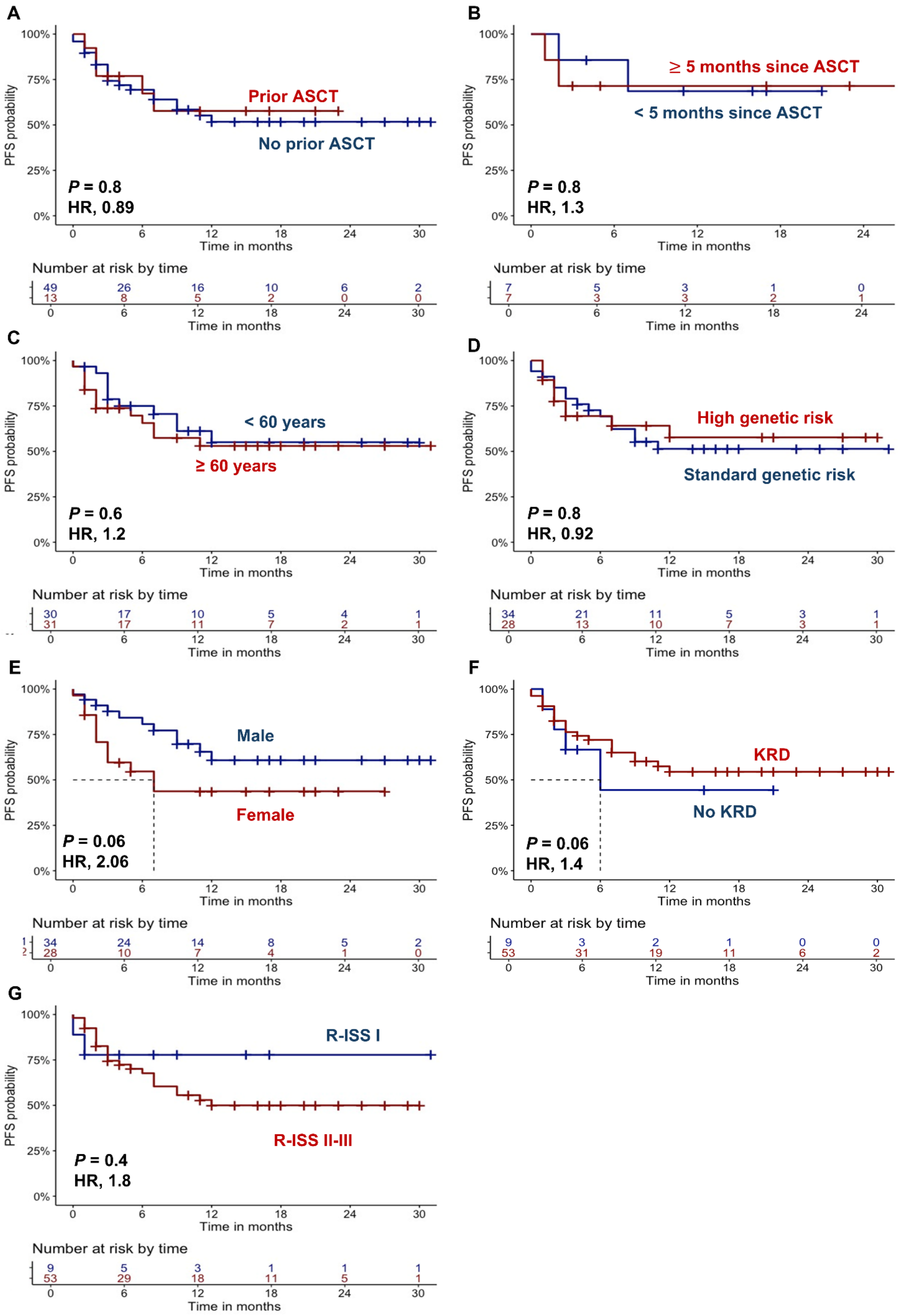
A
PFS probability
100%
75%
50%
25%
0%
Prior ASCT
No prior ASCT
P = 0.8
HR, 0.89
Time in months
Number at risk by time
49 26 16 10 6 2
13 8 5 2 0 0
0 6 12 18 24 30
B
≥ 5 months since ASCT
< 5 months since ASCT
P = 0.8
HR, 1.3
Number at risk by time
7 5 3 1 0
7 3 3 2 1
0 6 12 18 24
C
< 60 years
≥ 60 years
P = 0.6
HR, 1.2
Number at risk by time
30 17 10 5 4 1
31 17 11 7 2 1
D
High genetic risk
Standard genetic risk
P = 0.8
HR, 0.92
Number at risk by time
34 21 11 5 3 1
28 13 10 7 3 1
E
Male
Female
P = 0.06
HR, 2.06
Number at risk by time
34 24 14 8 5 2
28 10 7 4 1 0
F
KRD
No KRD
P = 0.06
HR, 1.4
Number at risk by time
9 3 2 1 0 0
53 31 19 11 6 2
G
R-ISS I
R-ISS II-III
P = 0.4
HR, 1.8
Number at risk by time
9 5 3 1 1 1
53 29 18 11 5 1

Supplementary Figure 1. Progression-free survival (PFS) in functional high risk multiple myeloma patients treated with second-line therapies. PFS is shown for patients after stratification for (A) prior autologous stem cell transplantation (ASCT), (B) relapse/refractoriness within 5 months since ASCT, (C) age ≥ 60 years old, (D) high genetic risk disease, (E) sex, (F) carfilzomib-lenalidomide-dexamethasone (KRD) administration, or (G) disease stage. A $P < 0.05$ was considered statistically significant. HR, hazard ratio.

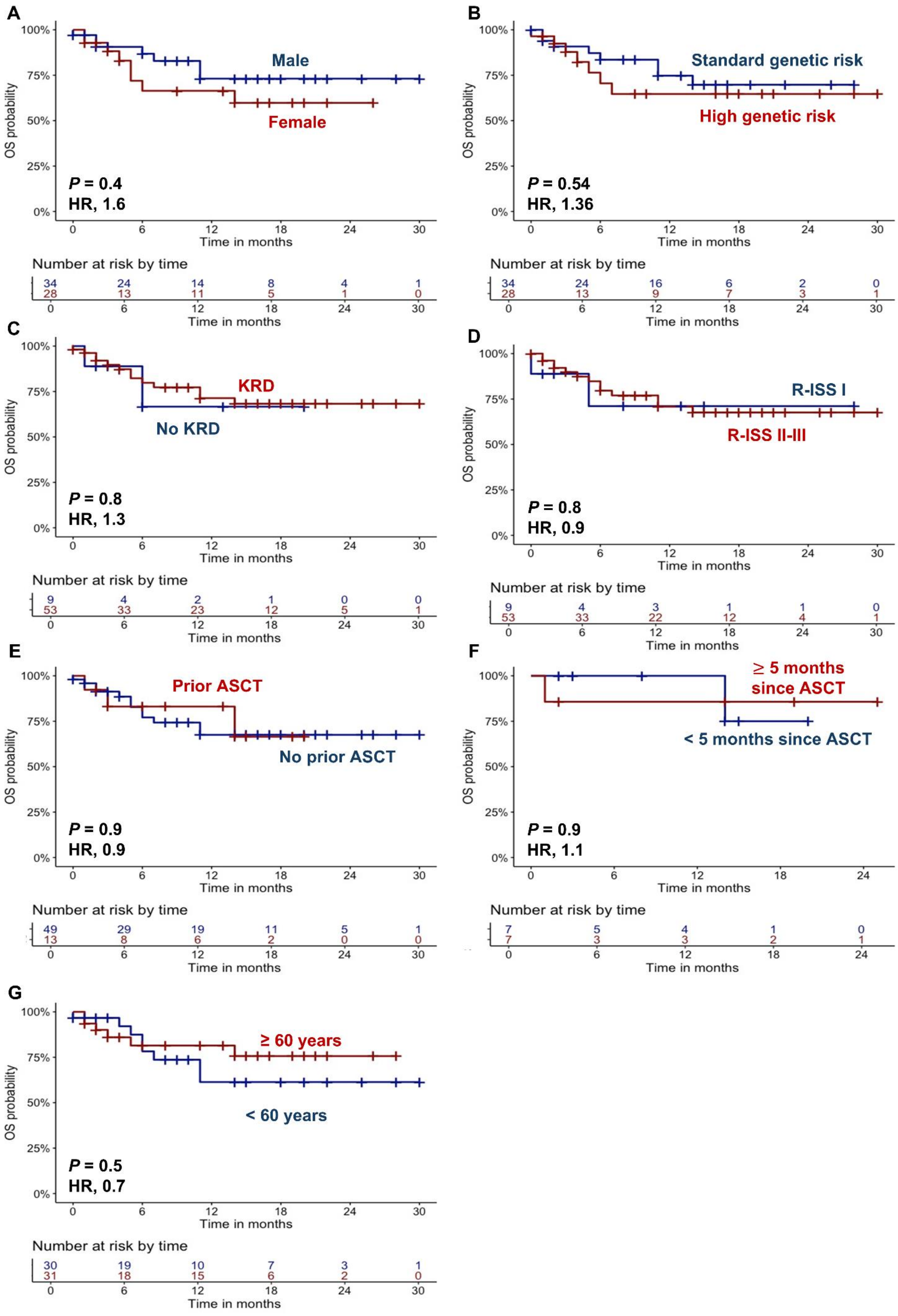
A
OS probability
100%
75%
50%
25%
0%
Male
Female
P = 0.4
HR, 1.6
Time in months
Number at risk by time
34 24 14 8 4 1
28 13 11 5 1 0
B
Standard genetic risk
High genetic risk
P = 0.54
HR, 1.36
Number at risk by time
34 24 16 6 2 0
28 13 9 7 3 1
C
KRD
No KRD
P = 0.8
HR, 1.3
Number at risk by time
9 4 2 1 0 0
53 33 23 12 5 1
D
R-ISS I
R-ISS II-III
P = 0.8
HR, 0.9
Number at risk by time
9 4 3 1 1 0
53 33 22 12 4 1
E
Prior ASCT
No prior ASCT
P = 0.9
HR, 0.9
Number at risk by time
49 29 19 11 5 1
13 8 6 2 0 0
F
≥ 5 months
since ASCT
< 5 months since ASCT
P = 0.9
HR, 1.1
Number at risk by time
7 5 4 1 0
7 3 3 2 1
G
≥ 60 years
< 60 years
P = 0.5
HR, 0.7
Number at risk by time
30 19 10 7 3 1
31 18 15 6 2 0

Supplementary Figure 2. Overall survival (OS) in functional high risk multiple myeloma patients treated with second-line therapies. OS is shown for patients after stratification for (A) sex, (B) high genetic risk disease, (C) carfilzomib-lenalidomide-dexamethasone (KRD) administration, (D) disease stage, (E) prior autologous stem cell transplantation (ASCT), (F) relapse/refractoriness within 5 months since ASCT, or (G) age ≥ 60 years old. A $P < 0.05$ was considered statistically significant. HR, hazard ratio.
